# Novel Rodent Arterivirus Detected in the Brazilian Amazon

**DOI:** 10.3390/v15051150

**Published:** 2023-05-11

**Authors:** Thito Y. Bezerra da Paz, Leonardo H. Almeida Hernández, Sandro Patroca da Silva, Fábio Silva da Silva, Bruno C. Veloso de Barros, Livia M. Neves Casseb, Ricardo J. de Paula Souza e Guimarães, Pedro F. da Costa Vasconcelos, Ana C. Ribeiro Cruz

**Affiliations:** 1 Parasite Biology in the Amazon Region Graduate Program, Pará State University, Belém 66087-670, Brazil; 2 Department of Arbovirology and Hemorrhagic Fevers, Evandro Chagas Institute, Health and Environment Surveillance Secretariat, Ministry of Health, Ananindeua 67030-000, Brazil; 3 Geoprocessing Laboratory, Evandro Chagas Institute, Health and Environment Surveillance Secretariat, Ministry of Health, Ananindeua 67030-000, Brazil

**Keywords:** *Arteriviridae*, arterivirus, RNA-seq, RNA viruses, Sigmodontinae

## Abstract

As part of a continuous effort to investigate the viral communities associated with wild mammals at the human–animal interface in an Amazonian metropolitan region, this study describes the detection of a novel rodent-borne arterivirus. A sample containing pooled organs of *Oecomys paricola* was submitted to RNA sequencing, and four sequences taxonomically assigned as related to the *Arteriviridae* family were recovered, corresponding to an almost complete genome of nearly 13 kb summed. In the phylogenetic analysis with the standard domains used for taxa demarcation in the family, the tentatively named Oecomys arterivirus 1 (OAV-1) was placed within the clade of rodent- and porcine-associated viruses, corresponding to the *Variarterivirinae* subfamily. The divergence analysis, based on the same amino acid alignment, corroborated the hypothesis that the virus may represent a new genus within the subfamily. These findings contribute to the expansion of the current knowledge about the diversity, host and geographical range of the viral family. Arterivirids are non-human pathogens and are usually species-specific, but the susceptibility of cell lines derived from different organisms should be conducted to confirm these statements for this proposed new genus in an initial attempt to assess its spillover potential.

## 1. Introduction

The known diversity of the virosphere increases as high throughput sequencing technologies become more affordable and widespread, allowing metagenomic studies for viral surveillance to thrive [1]. Taking these advances into account, the International Committee on Taxonomy of Viruses (ICTV) incorporated metagenomics as a key source of biological information for viral classification. Many viral taxa were expanded and reorganized following the discovery of new viral agents detected exclusively using this approach [2,3]. The *Arteriviridae* family, order *Nidovirales*, which has had a sixfold increase in its species content since the 2014 ICTV release, was thoroughly reorganized based mainly on criteria relating to genomic information [4]. Currently, the family comprises six subfamilies, 13 genera, 11 subgenera, and 23 species. From these, seven species, from four genera of the *Variarterivirinae* and *Heroarterivirinae* subfamilies, are associated with rodents [5].

Most viruses in this family lack experimental characterization. However, based on those more intensely studied, they are assumed to present spherical, pleomorphic, enveloped virions ranging from 50 to 74 nm in diameter, containing a positive-sense, single-stranded and multi-cistron RNA genome of 12.7–15.7 kb. The genomic organization of the family slightly varies in the ORF’s content but conserves characteristic features and domains among all species. Five domains located in the replicase ORFs 1a (3C-like protease-3CLpro) and 1b (nidovirus RdRp-associated nucleotidyltransferase-NiRAN; RNA-dependent RNA polymerase-RdRp; Zn-binding domain-ZBD; and superfamily 1 helicase-HEL1) are the standard markers for the phylogenetic inferences and genetic distance indexes used for taxonomic demarcation within the family [5].

Arterivirids, including those not yet assigned to any species, are non-human mammalian viruses with a wide range of hosts, including horses, porcines, rodents, simians, shrews, possums, bank voles and hedgehogs, and the family is believed to be even more diverse [5,6,7,8,9]. Most of the first arteriviruses described are pathogens of significant veterinary importance, as equine arteritis virus (EAV), porcine reproductive and respiratory syndrome virus 1 and 2 (PRRSV-1/PRRSV-2) and simian hemorrhagic fever virus (SHFV), which produce overt disease in horses, domestic pigs and non-human primates, respectively. Lactate dehydrogenase elevating-virus (LDV) is an exception, being associated with subclinical infections in mice [5].

Among the most recently described arteriviruses, wobbly possum disease virus (WPDV) and hedgehog arterivirus (HhAV-1) were imputed as causative agents of disease in captive possums and wild hedgehogs, respectively [6,8]. All the recently described rodent arteriviruses have Asian, African or European origin, sampled from individuals collected in China, Cameroon, Tanzania, Mozambique and Ukraine, mainly in metagenomic studies [7,9,10]. This report describes an almost complete genome of a novel rodent arterivirus, which might represent a new genus of the *Arteriviridae* family, the first, to our knowledge, detected in Brazil, and the most complete genomic record in Latin America [11].

## 2. Materials and Methods

### 2.1. Sample Collection

As part of a larger surveillance effort to investigate the viral communities associated with wild mammals at the human–animal interface, covering areas with phytophysionomical similarities and presenting land use changes, tissue samples were collected from wild mammals in Santa Bárbara do Pará, a municipality located in the Belém metropolitan mesoregion of Pará State, Brazil. The captures, sample collection and the further laboratorial proceedings were authorized by the Ethics Commission on Animal Use at the Evandro Chagas Institute under protocols no. 21/2014 and 41/2019.

**Figure 1:**
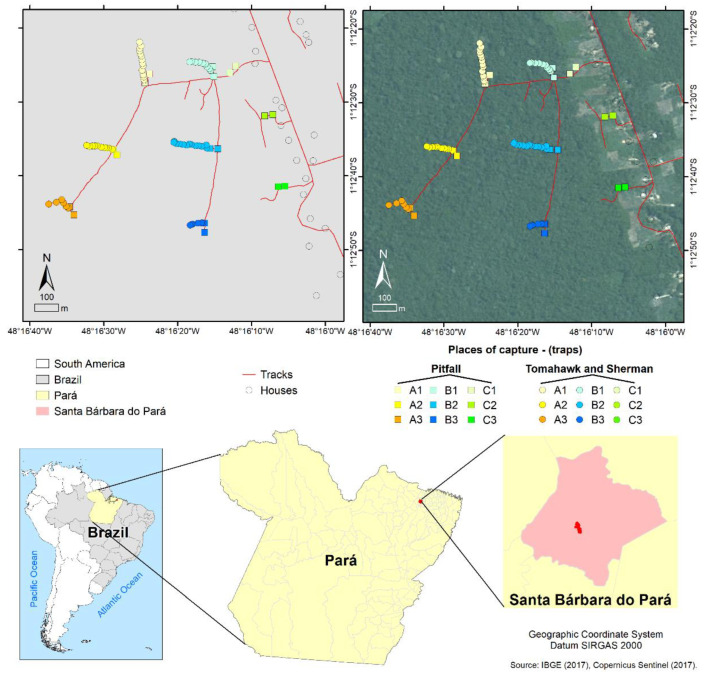
Map of the collection area in the Expedito Ribeiro village in Santa Bárbara do Pará municipality (Pará state, Brazil) and its location in the Brazilian territory. Traps were placed in the open field surrounding the human habitations (A1–A3), the forest fragment border (B1–B3) and its most interior region (C1–C3). The IBGE (2017) [12] and Copernicus Sentinel (2017) [13] databases were consulted to prepare the maps.

Animals were captured in the forest area in the surroundings of human habitations and their annexed agricultural areas. Tomahawk and Sherman traps were positioned alongside a trail, covering the forest fragment border and its most interior region, and in the open field surrounding human habitations (Figure 1). Two expeditions were conducted in October 2014 and April 2015, resulting in the capture of 39 mammals: 18 marsupials, 12 rodents and nine chiropterans.

After the collection of blood and serum samples, the animals were anesthetized with Zoletil^®^ 50 via an intramuscular route, followed by euthanization applying cervical dislocation prior to the harvesting of tissue samples from the spleen, lymph nodes, heart and lungs, which were submitted to a first pooling process into a single cryotube per animal. Samples were transported in liquid nitrogen to the Department of Arbovirology and Hemorrhagic Fevers at the Evandro Chagas Institute (Ananindeua, Brazil), and maintained at −80 °C until downstream processing. A first subset of five pooled tissue samples of rodents, morphologically identified as *Oecomys* sp., with no apparent signs of illness, was arbitrarily chosen to initiate the processing for sequencing and metagenomic analysis.

### 2.2. RNA Extraction and cDNA Synthesis

The samples from five rodent specimens collected in April 2015 were submitted to a second pooling process into a single sample, which was the source material for the sequencing, containing small fragments of the above-mentioned tissues from each animal and submitted to RNA extraction. A 5 mm tungsten bead and 600 µL of 1-Thioglycerol/Homogenization Solution were added to the sample, followed by mechanical disruption of the fragments for 2 min at 25 Hz in a TissueLyser II system (Qiagen, Hilden, Germany). RNA extraction was performed with a Maxwell^®^ 16 LEV simplyRNA Tissue Kit (Promega, Madison, WI, USA) in the Maxwell^®^ 16 System (Promega) according to the manufacturer’s protocol. SuperScript^TM^ VILO^TM^ Master Mix (Thermo Fischer Scientific, Waltham, MA, USA) was used for the first strand synthesis of complementary DNA (cDNA) and the NEBNext^®^ mRNA Second Strand Synthesis Module (New England BioLabs, Ipswich, MA, USA) for the second strand synthesis.

### 2.3. Sequencing and Sequence Assembly

The cDNA library for shotgun sequencing was prepared using the Nextera XT DNA Library Preparation Kit (Illumina, San Diego, CA, USA). Quantification and fragmentation level assessment were performed using a Qubit 2.0 Fluorometer (Thermo Fischer Scientific) and 2100 Bioanalyzer Instrument (Agilent Technologies, Santa Clara, CA, USA), respectively, prior to sequencing on the NextSeq 500 System (Illumina) applying the NextSeq 500/550 Mid Output (300 cycles), with a paired-end (2 × 150 pb) protocol.

Raw reads corresponding to adaptors and with undetermined bases were removed by Trim Galore v.0.4.5 [14], ribosomal RNA by SortMeRNA v.2.1 [15], and redundant sequences by CD-HIT [16]. The remaining reads were De Novo assembled by IDBA-UD (k-mers 20, 40, 60, 80, and 100) and MEGAHIT (k-mers 21, 29, 39, 59, 79, 99, 119, and 141) [17,18]. Contigs were then aligned against the non-redundant protein database by DIAMOND [19] with a 10^−3^ e-value threshold, and inspected with MEGAN6 [20], in which five contigs were assigned as related to the *Arteriviridae* family. The contigs from both assemblers were imported to Geneious Prime [21] and aligned against the reads to detect eventual gaps. The aligned reads were De Novo assembled using Geneious native assembler resulting in four final sequences.

The raw metagenomic data are available at SRA/NCBI database under the accession number SRR23801058, BioProject PRJNA882858. The final sequences are available under the accession numbers OQ686610 to OQ686613.

### 2.4. Phylogenetic Reconstruction and Divergence Analysis

The contigs’ ORFs were predicted and translated, and the ORF1a and ORF1b amino acid sequences domains were annotated using the InterProScan [22] extension on Geneious Prime. The 3CLpro, Niran, RdRp, ZBD and HEL1 domains were extracted, concatenated and realigned using the Clustal Omega [23] and MAFFT [24] extension on Geneious Prime to the alignment provided at the Resources page in the ICTV report chapter [25], comprising the concatenated domains of the 23 currently recognized *Arteriviridae* species. The resulting amino acid alignment was used to generate a *Maximum Likelihood* phylogenetic reconstruction with 1000 bootstrap iterations applying the LG + F + I + G4 substitution model on IQ-TREE 2 [26], with the previous estimation of the phylogenetic signal. An additional phylogenetic analysis was performed using the amino acid alignment of the predicted sequence of polyprotein 1b (pp1b) under the same parameters. The p-distances corresponding to the alignment of the five concatenated domains were imported from MEGA X [27] and pairwise patristic distances (PPD) were calculated using the MrBayes [28] extension. The p-distance values were used to generate a boxplot graph of the intergroup and intragroup amino acid distances of the *Variarterivirinae* and *Simarterivirinae* subfamilies in comparison with the distance between their members and the novel arterivirus.

## 3. Results

**Figure 2:**
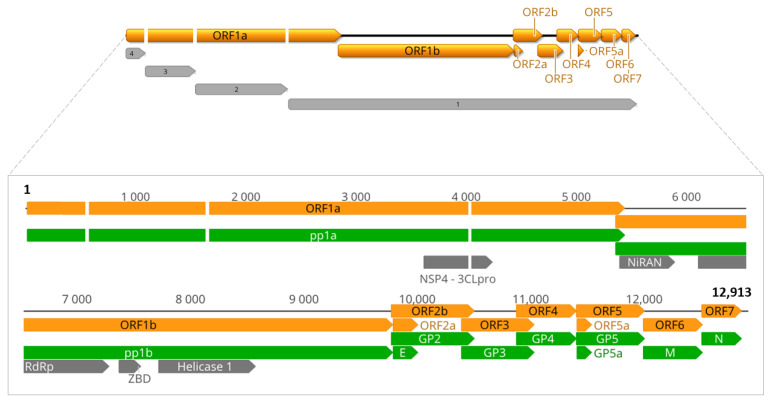
Oecomys arterivirus 1 (OAV-1) partial genome. The sequences above are represented as contiguous for illustrative purposes, highlighting the genome organization. The non-sequenced regions are identified as blank gaps. Contigs are numbered from 1 to 4. In the zoomed sequence, domains in gray were concatenated for the phylogenetic inference. 3CLpro, 3C-like protease; NiRAN, nidovirus RdRp-associated nucleotidyltransferase; RdRp, RNA-dependent RNA polymerase; ZBD, Zn-binding domain; Helicase 1, super-family 1 helicase; E, envelope protein; GP2-GP5a, envelope glycoproteins; N: nucleocapsid protein.

An almost complete genome was retrieved, comprising four final contigs assigned as related to known arterivirids. The ordination of the sequences revealed the classic genome organization of the *Arteriviridae* family members as illustrated in Figure 2, altogether comprising approximately 12.9 kb, which is inside the genome size range of the family. Metrics regarding the sequencing and the coverage of the final contigs are provided in Tables S2 and S3. Maximum nucleotide and amino acid identities of 38.38% and 45.47%, respectively, were obtained for PRRSV-1 (M96262), and comparable values were observed for all *Variarterivirinae* subfamily members. A comparison of the identities between the translated ORFs of OAV-1 and the recognized variarterivirins against PRRSV-1 is provided in Table S3 and Figure S2.

ORF1a and ORF1b, the two major ORFs of arterivirid genomes, which express the non-structural proteins, and the minor ORFs for the structural proteins next to the 3′ terminal region (envelope, E; glycoproteins, GP2 to GP5; membrane, M; and nucleocapsid; N), could be predicted. Besides the domains used for phylogenetic inference, three cysteine-protease domains of NSP1 and NSP2, characteristic for arteriviruses, were identified in the predicted sequence of polyprotein 1a by InterProScan search, along with domains characteristic for NSP4 and NSP7.

Additionally, the complete mitochondrial genome of the host was recovered, and a BLAST search of the *CytB* gene, deposited under accession number OQ623637, showed a 99.38% identity with *Oecomys paricola*. The virus was tentatively named after the host genus as Oecomys arterivirus 1 (OAV-1).

**Figure 3:**
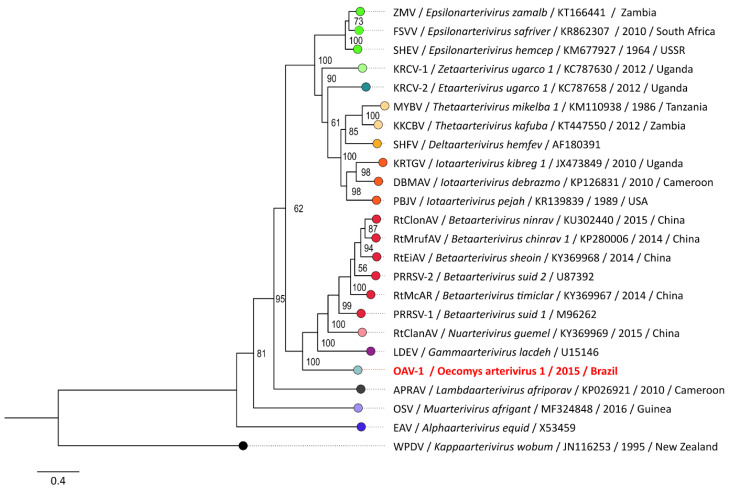
Phylogenetic inference of the currently recognized species of *Arteriviridae* family based on the concatenated sequences of the 3CLpro, NiRAN, RdRP, ZBD and HEL1 domains. Genera are distinguished by color and can be identified in the species name. The sequence of this study is highlighted in red.

**Figure 4:**
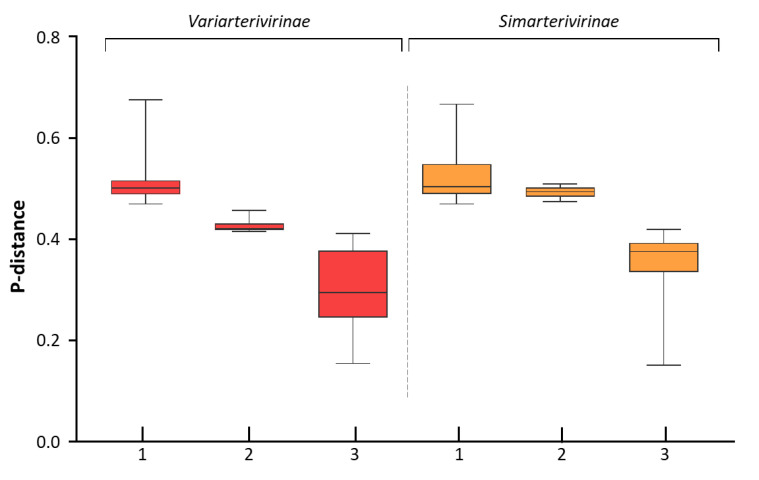
Boxplot graph showing the amino acid distance range between the *Variarterivirinae* and *Simarterivirinae* subfamilies and arterivirids outside their taxa, or intergroup distances (1), distances between them and OAV-1 (2), and distances within each subfamily, or intragroup distances (3), based on the same alignment used to generate the phylogenetic inference.

**Figure A1:**
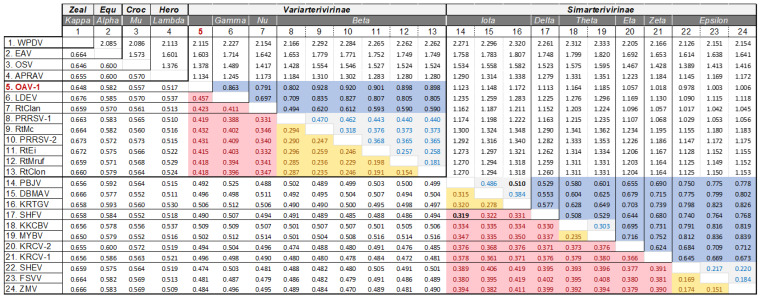
P-distance (inferior left) and PPD (superior right) calculated based on the five concatenated domains alignment used for the phylogenetic inference. Cells with values under the estimated threshold are highlighted for subfamilies (red/blue) and genera (yellow/gray). Outlier values are written in bold font. Genera and some subfamilies are identified by their prefixes.

A phylogenetic signal of 96.5% was estimated for the dataset used to generate the phylogenetic inference depicted in Figure 3. The novel arterivirus was placed with a high support bootstrap value at the most basal branch in the clade of porcine and rodent arterivirids, corresponding to the *Variarterivirinae* subfamily clade, which is the most proximal taxon as demonstrated in the boxplot graph of amino acid distances presented in Figure 4. The p-distance and PPD indexes calculated for the alignment and highlighted taxa demarcations are provided in Figure A1. The additional phylogenetic analysis based on the pp1b alignment is available in the Supplementary Materials (Figure S1). Despite minor discordances in the tree topology, similar results were obtained regarding the placement of OAV-1 in the *Variarterivirinae* clade.

The intergroup and intragroup distances represent the overall level of amino acid divergence between the selected taxon and the other representants of the family, and within the subfamily, respectively. The intergroup distances are above a minimum p-distance value of 0.470 for the *Variarterivirinae* and *Simarterivirinae* subfamilies, which possess the largest number of species and are the only ones that harbor more than one species.

Although the mean distance between OAV-1 and members of the *Variarterivirinae* subfamily slightly surpasses maximum intragroup distance for the subfamily, the taxon is the most related to the virus, and the distance values between OAV-1 and the variarterivirins are completely out of the intergroup range. Furthermore, as for comparison, the distances between OAV-1 and four of the eight *Variarterivirinae* species are equal or lower than those found between members of distinct genera in the *Simarterivirinae* subfamily.

## 4. Discussion

Due to the abundant biodiversity present in the Amazon biome, its viral diversity is also assumed to reach considerably high levels. However, much of this diversity remains unknown since research efforts are still insufficient to cover its massive territory [29,30]. The recovery of an almost complete genome of a novel rodent arterivirus through high throughput sequencing in a metagenomic approach contributes to the expansion of the knowledge about the diversity, host range and geographic distribution of the *Arteriviridae* family, which many studies have shown to be much larger than previously known, and are predicted to increase as research on viral discovery advances [4,6,8,31].

Although the recovered OAV-1 sequences correspond to a draft genome, presenting gaps and low coverage in some regions, which may preclude the detection of residual sequencing errors, these are unlikely to compromise the overall conclusions of this study. The OAV-1 is highly divergent on both nucleotide and amino acid levels, providing evidence that it might be part of a singular clade of arteriviruses, which might be exclusive to the Americas. Its inclusion in a basal and highly supported branch on the phylogenetic inference, in addition to the distance analysis, provides strong evidence to propose the inclusion of the virus as a divergent representant of a new genus in the *Variarterivirinae* subfamily, which includes important veterinary pathogens.

Additionally, some details on tree topology slightly differ from those presented in the ICTV report chapter [5], which was expected since the presented *diversity partitioning by hierarchical clustering* (DEmARC) analysis included additional nidovirus sequences and substitution model estimation was performed individually for each domain, as reported. The discovery of novel divergent arteriviruses may occasionally challenge the current taxonomic classification and imply a need for changes in the threshold values for taxa demarcation as new sequences become available.

Arterivirids are non-human pathogens and are usually species-specific. However, their importance as livestock and wildlife pathogens cannot be neglected [32]. Spillover events may occur in natural environments, most probably between taxonomically related hosts, and remain undetected, since efforts to ecologically characterize members of this family are scarce. Recently, HhAV-1 was imputed as the causative agent of fatal encephalitis in hedgehogs admitted to a center for wildlife rehabilitation, and it was assumed to have been carried, most likely, by asymptomatic hedgehogs; however, the possibility that the virus could have been introduced by an asymptomatic animal from a different species held in the same facility was also considered [8].

Similar events, in which captive primates of different species developed simian hemorrhagic fever (SFH), have occurred multiple times in the past decades [33]. Indeed, some of the previous outbreaks of SFH credited to SHFV were posteriorly attributed to simian hemorrhagic encephalitis virus (SHEV) and Pebjah virus (PBJV), leading to the hypothesis of cross-species transmission due to contact with the natural hosts of simian arteriviruses and demonstrating that the ability to infect new hosts may be a common trait within this clade [34]. For instance, the risk of interspecies transmission of simian arteriviruses to humans is a recurrent concern [35] with recent evidence pointing to a certain level of permissibility of human monocytes to SHFV in vitro, highlighting the importance of understanding the pathogenesis and transmission dynamics of the several recently discovered arterivirids [36].

Furthermore, the close phylogenetic relationship between the porcine and rodent arteriviruses may make this novel virus worthy of attention in further surveillance activities. Although there is no evidence for direct transmission of these viruses from rodents to domestic animals, interspecies transmission of a rodent-borne arterivirus to wild boars was hypothesized to have been the route from which PRRSV-1/2 independently evolved to infect domestic pigs in central Europe and North America [37].

Considered as an Amazonian species, the Brazilian arboreal rice rat, *Oecomys paricola*, was identified as the host of OAV-1. Formally, the taxon is considered as a single species, although the most recent evidence supports its reorganization as a species complex [38]. South America accounts for about 23% of the mammalian diversity in the world, and rodents are among the most prolific and diverse orders [39]. For this reason, several studies have focused on the characterization of their virome; however, most of these have concentrated on North America and Asia [10,40,41,42], with less efforts made in the field in Central and South America, and, especially, in the Amazon region. A single study provided genomic evidence for the circulation of arteriviruses in rodents of the *Proechimys guyanensis* species in French Guyana; however, only the raw sequencing data was published [11].

The local economy of the small rural properties in the surroundings of the forest where the specimens of *O. paricola* were captured includes swine farming and lacks the structural capacity to maintain a biosafe environment, apart from the local fauna [43]. Although the potential for infectious disease amplification in small scale animal husbandry is low, the proximity to the forest fragment may expose domestic animals to wildlife pathogens [44].

Therefore, increased contact between domestic and wild species is a key factor favoring viral spillover. Despite the particularities observed for cross-species transmission of arteriviruses, RNA viruses in general have high genome plasticity, which could facilitate their adaptation to new hosts [35,45]. For this reason, susceptibility to viral infection by OAV-1 of cell lines derived from different organisms should be assessed to confirm these statements for this proposed new genus in an initial attempt to estimate its spillover potential [31].

Besides PRRSV-1, EAV and LDV, most members of the family were detected or isolated in Old World countries, including all simian and other rodent-borne arteriviruses described [9,32]. Continuous sampling of distinct species of the genus, which is considerably speciose [46], along with other mammalian species native to the region in further surveillance studies, is essential to unravel the diversity and coevolutionary patterns of the *Arteriviridae* family in the Amazon and, in a broader context, within the American continent.

## Data Availability

The final sequences and raw sequencing data are deposited in GenBank under accession number OQ686610 to OQ686613, and SRR23801058.

## Supplementary Materials

The following supporting information can be downloaded at: https://www.mdpi.com/article/10.3390/v15051150/s1, Figure S1. Phylogenetic reconstruction based on the polyprotein 1b predicted sequence alignment; Table S1. Number of reads in each step of analysis; Table S2. Coverage metrics for each contig; Table S3. Identities of the translated sequences of each ORF from OAV-1 and the recognized variarterivirins against PRSSV-1; Figure S2. Graph of the identities of the translated sequences of each ORF from OAV-1 and the recognized variarterivirins against PRSSV-1.
