# Supplementary material for: Novel Rodent Arterivirus Detected in the Brazilian Amazon
Source: Viruses. 2023 May 11;15(5):1150. doi: 10.3390/v15051150 (PMC10222503; doi:10.3390/v15051150)
Supplement: Supplementary file 1 [file viruses-15-01150-s001.zip › Supplementary Figure.pdf]

Supplementary Figure

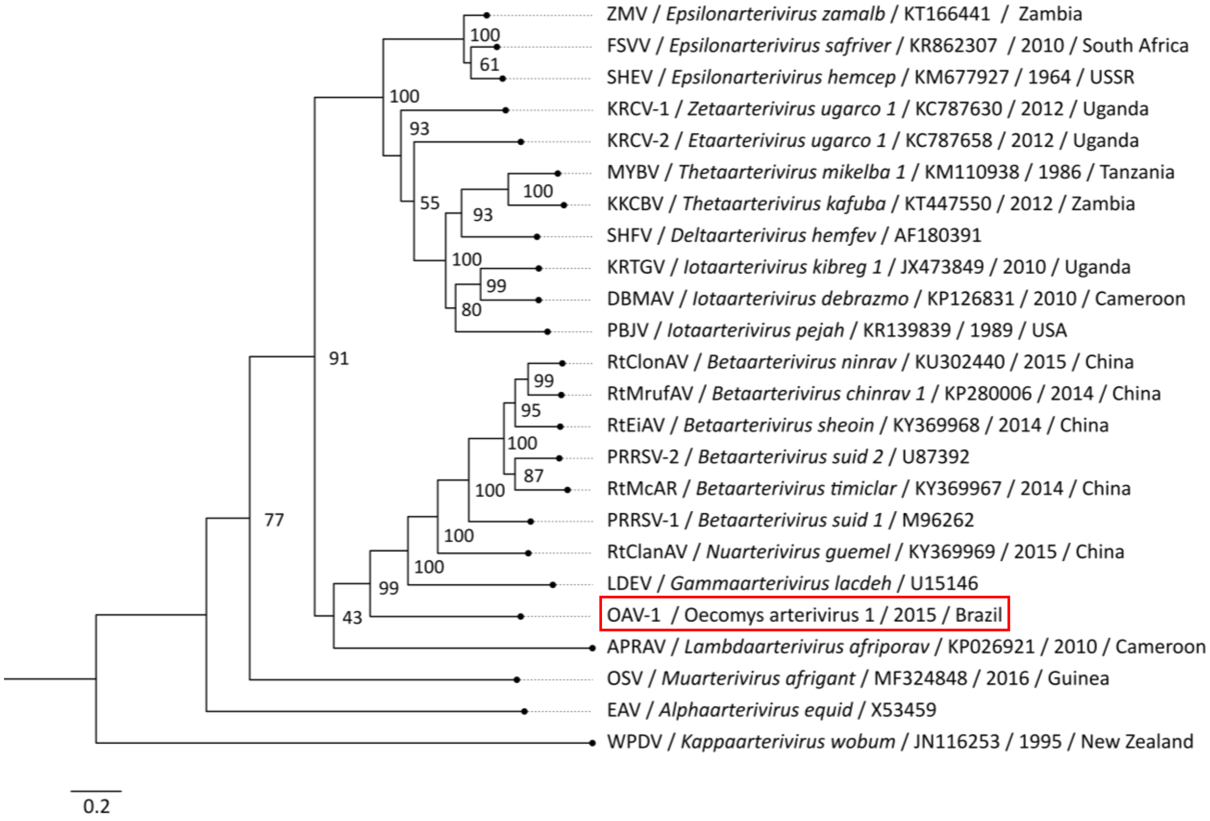

Figure S1. Phylogenetic reconstruction based on the polyprotein 1b predicted sequence alignment.
